# Diaphragmatic electromyography during a spontaneous breathing trial to predict extubation failure in preterm infants

**DOI:** 10.1038/s41390-022-02085-w

**Published:** 2022-05-06

**Authors:** Emma E. Williams, Fahad M. S. Arattu Thodika, Imogen Chappelow, Nicole Chapman-Hatchett, Theodore Dassios, Anne Greenough

**Affiliations:** 1 Department of Women and Children’s Health, School of Life Course Sciences, Faculty of Life Sciences and Medicine, King’s College London, London, UK; 2 Neonatal Intensive Care Centre, King’s College Hospital NHS Foundation Trust, London, UK; 3 Asthma UK Centre for Allergic Mechanisms, King’s College London, London, UK; 4 NIHR Biomedical Research Centre based at Guy’s and St Thomas’ NHS Foundation Trust and King’s College London, London, UK

## Abstract

**Background:**

Premature attempts at extubation and prolonged episodes of ventilatory support in preterm infants have adverse outcomes. The aim of this study was to determine whether measuring the electrical activity of the diaphragm during a spontaneous breathing trial (SBT) could predict extubation failure in preterm infants.

**Methods:**

When infants were ready for extubation, the electrical activity of the diaphragm was measured by transcutaneous electromyography (EMG) before and during a SBT when the infants were on endotracheal continuous positive airway pressure.

**Results:**

Forty-eight infants were recruited (median (IQR) gestational age of 27.2 (25.6–30.4) weeks). Three infants did not pass the SBT and 13 failed extubation. The amplitude of the EMG increased during the SBT [2.3 (1.5–4.2) versus 3.5 (2.1–5.3) µV; *p* < 0.001]. In the whole cohort, postmenstrual age (PMA) was the strongest predictor for extubation failure (area under the curve (AUC) 0.77). In infants of gestational age <29 weeks, the percentage change of the EMG predicted extubation failure with an AUC of 0.74 while PMA was not associated with the outcome of extubation.

**Conclusions:**

In all preterm infants, PMA was the strongest predictor of extubation failure; in those born <29 weeks of gestation, diaphragmatic electromyography during an SBT was the best predictor of extubation failure.

**Impact:**

- Composite assessments of readiness for extubation may be beneficial in the preterm population.
- Diaphragmatic electromyography measured by surface electrodes is a non-invasive technique to assess the electrical activity of the diaphragm.
- Postmenstrual age was the strongest predictor of extubation outcome in preterm infants.
- The change in diaphragmatic activity during a spontaneous breathing trial in extremely prematurely born infants can predict subsequent extubation failure with moderate sensitivity and specificity.

## Introduction

Prematurely born infants who develop respiratory distress at birth often require mechanical ventilation because of increasing respiratory failure.^1^ A prolonged duration of invasive ventilation has been associated with the development of bronchopulmonary dysplasia and neurodevelopmental impairment.^2,3^ Conversely, early extubation and subsequent need for reintubation has been linked with prolonged length of neonatal stay and increased mortality.^4,5^ As one-third of preterm neonates fail extubation,^5–10^ with the rate of failure due to respiratory causes increasing up to 70% in those born extremely prematurely,^11–13^, it is important that extubation failure can be more accurately predicted. Methods to accurately predict the readiness of preterm infants for extubation have been reported to be either of low predictive ability or methodologically cumbersome.^6,7,14,15^ Therefore, the decision to extubate is often based on subjective clinical judgement with variability in extubation practices.^15,16^ Furthermore, the duration of observation following extubation is extremely variable in the population, thus making the definition of extubation success often inconsistent and difficult to compare between studies.^11,13^ Successful extubation is dependent on the balance between the respiratory load, respiratory muscle strength, and respiratory drive.^17,18^ Respiratory drive and the ability of the diaphragm to respond to an increased respiratory load are major determinants of extubation success in infants.^8,19,20^

The electrical activity of the diaphragm (Edi) is a quantifiable measure of neural respiratory drive and the diaphragmatic response to inspiratory load.^21^ Pre-extubation assessment of the Edi, measured by an oesophageal catheter, as a predictor of successful extubation has been utilised in adult and paediatric intensive care.^22–24^ Furthermore, diaphragmatic activity as measured by transcutaneous electrodes in infants and children has been shown to be higher pre and post extubation in those subsequently failing extubation.^25^ The Edi in the neonatal population may also be measured using non-invasive surface electromyography (EMG). A previous study of EMG in ventilated infants, however, did not show a significant difference in the results between those infants who were successfully extubated or failed extubation.^26^ In that study, however, the EMG was measured during invasive ventilatory support, which could have suppressed the respiratory drive and hence any difference in diaphragmatic function would not have been observed. The use of a spontaneous breathing trial (SBT) has been reported to be 97% sensitive but of low (40%) specificity in predicting readiness for extubation,^27^ making use of an SBT alone not routinely recommended in preterm infants given the inaccuracy in predicting extubation failures^28,29^ along with reports of clinical instability. We thus hypothesised that a combined measurement of the Edi during an SBT would increase the specificity of the assessment. Hence, the aim of this study was to determine whether measuring the diaphragmatic EMG during an SBT would be useful in predicting extubation failure in infants born prematurely, particularly those born very prematurely, that is <29 weeks of gestational age.

## Methods

A prospective study was undertaken in the neonatal intensive care unit at King’s College Hospital NHS Foundation Trust between 22 June 2020 and 10 July 2021. The South West - Central Bristol Research Ethics Committee and Health Research Authority approved the study (REC reference 20/SW/0062) and parents gave written, informed consent for their infants to take part in the study. Infants born prior to 37 completed weeks of gestation were eligible for recruitment if receiving invasive mechanical ventilation. Infants with congenital lung or diaphragmatic anomalies were excluded. Ventilatory support was provided by the SLE6000 ventilator (SLE Ltd, Croydon, UK) via shouldered Cole’s endotracheal tubes. Patient-triggered volume-targeted ventilation, with target tidal volumes of 5–7 ml/kg, was used. Positive end expiratory pressures (PEEPs) of 5–6 cmH_2_O were used for all infants, as per the Unit’s protocol. Low-dose morphine infusion (5–10 mcg/kg/h) was used for sedation in infants who were thought to be in pain, irrespective of their gestational age. Infants were extubated when the morphine infusion was stopped or was at a dose of 5 mcg/kg/h.

Infants were studied once deemed ready for extubation by the clinical team if they had acceptable blood gases (pH > 7.25, partial pressure of carbon dioxide (PaCO_2_) of <8.5 kPa), were requiring a fraction of inspired oxygen (FiO_2_) <0.4, and had a breathing rate above the back-up rate of the ventilator. Infants were extubated as per the decision of the clinical team onto non-invasive ventilatory support. Non-invasive respiratory support consisted of continuous positive airway pressure (CPAP) or high-flow oxygen therapy, according to clinician preference. The two modes of non-invasive support were considered non-inferior to each other.^30^ If infants required re-intubation within the subsequent 48 h, this was recorded as a failed extubation. Secondary analysis from the SUPPORT trial found that, among infants failing extubation within 5 days, 75% of those infants required reintubation within 2 days, hence we chose 48 h as the criteria for extubation failure.^4^ Criteria for reintubation were respiratory acidosis on blood gas analysis (pH < 7.25 and PaCO_2_ > 8.5 kPa), apnoea requiring stimulation +/− bag-mask ventilation, or an FiO_2_ requirement of >0.6. The clinicians were blinded to the results of the SBT-EMG analysis.

### Spontaneous breathing trial

A SBT was performed by switching the infant from mechanical ventilatory support to endotracheal CPAP (Et-CPAP) at the same level of PEEP used during mechanical ventilation for a period of 10 min. Continuous heart rate and transcutaneous saturation monitoring was undertaken during the SBT. A failed SBT was recorded if there was bradycardia (heart rate <100) for over 15 s or a decrease in oxygen saturation (SpO_2_) to <85% despite a 15% increase in FiO_2_. If the infant failed the SBT, the test was stopped and mechanical ventilation was resumed. A 10-min SBT was chosen as per our unit protocol and as previously described in our studies.^31^ Furthermore, prolonged periods of Et-CPAP can induce diaphragmatic fatigue by breathing being unsupported through a narrow endotracheal tube.^32^

### Electromyography

The surface EMG of the diaphragm was recorded for 10 min before and 10 min during the SBT. The electrical activity of the diaphragm was measured at the cotside via transcutaneous EMG utilising a portable 16-channel digital physiological amplifier (Dipha-16, Inbiolab, Netherlands), which was connected to three surface electrodes (Kendall H59P cloth electrodes, Covidien, MA). One electrode was placed at the centre of the chest on the sternum, with the other two electrodes placed vertically in line with the nipples at both left and right costo-abdominal margins. Infants were nursed in the supine position during the study.

The physiological amplifier generated raw signals of diaphragmatic EMG at a sampling rate of 500 Hertz (Hz), which were then wirelessly transmitted to a portable bedside computer. Polybench software automatically processed and filtered the raw signals. The EMG data were imported into MATLAB for analysis, using the TMSi MATLAB Interface (Twente Medical Systems International, Oldenzaal, Netherlands). This software has been used previously by our research group for several studies in the neonatal population, in both term and preterm infants.^26,33,34^ The Polybench software has also be utilised to measure diaphragmatic activity in preterm infants in response to caffeine therapy.^35^

For each 10-min epoch (pre SBT and during SBT), the longest artefact-free trace was selected for analysis. This was done manually by the investigators by inspection of the recordings. The amplitude of the EMG was the average (mean) amplitude measured breath by breath over each 30 s artefact-free segment. Manual visualisation of the recordings to determine artefact-free periods was undertaken by two clinical research fellows blind to the outcome of extubation. Three 30 s periods were chosen and the average presented so that consistency between infants could be maintained; for example, in vigorous infants, some of the trace was required to be discarded due to signal artefact. The software automatically averaged and reported the mean EMG amplitude [microvolt (µV)] and mean EMG area under the curve (EMG_AUC_) [µV.S]. The mean amplitude was calculated to give a measure of the motor unit activity, but we also presented the mean area under the curve as an integration of this value over time. The percentage change in the electrical activity of the diaphragm from baseline to SBT was calculated to determine their relationship with subsequent extubation failure. The surface electrodes are susceptible to crosstalk from other muscles and movements. The signals are automatically filtered for noise; however, on visual inspection of the traces, small peaks <0.2 µV, in keeping with electrical activity of the heart rather than respiratory breaths, were excluded from the analyses.

### Demographic data

Demographic data were recorded including antenatal exposure to corticosteroids and magnesium sulphate, mode of delivery, gestational age at birth, birth weight, sex, administration of surfactant, methylxanthines and/or sedatives, postmenstrual age (PMA) and weight at the time of the study. The most recent blood gas prior to extubation was recorded including the pH, PaCO_2_ and haematocrit (Hct). Ventilator settings at the time of study were recorded, including the fraction of inspired oxygen (FiO_2_), mean airway pressure (MAP), expiratory tidal volume (Vte), and the back-up rate.

### Sample size

A previous study that used neurally adjusted ventilatory assist to measure the electrical activity of the diaphragm (EAdi) in preterm infants reported a standard deviation of 6.9 µV in the amplitude of the EAdi.^36^ The mean difference in the amplitude of the EAdi 30 min pre and post extubation, in the infants who were successfully extubated, was 6.8 µV. To detect such a difference in the EAdi amplitude with 80% power at the 5% significance level, assuming one-third of infants would fail extubation,^9^ 48 infants were required.

### Statistical analysis

Data were tested for normality using Shapiro–Wilk test and found not to be normally distributed, thus non-parametric tests were utilised. Chi-square test was used to determine whether the SBT result and outcome of extubation were statistically significant. Paired analysis was undertaken using Wilcoxon paired rank-sum test to determine the change in infant EMG amplitude and EMG_AUC_ during the SBT from baseline. To account for differences in the starting baseline for each infant, the percentage change from baseline to during the SBT of both the EMG amplitude and EMG_AUC_ were calculated. Mann–Whitney *U* test was then used to compare the EMG percentage (% EMG) changes between the two groups—those infants who failed extubation and those who succeeded. Binary logistic regression analysis determined the independent effect of % EMG change during the SBT in predicting extubation failure after accounting for differences between the two groups (level of significance *p* < 0.10). The performance of the electrical activity of the diaphragm during the SBT and PMA at time of study in predicting extubation failure were assessed using receiver operator characteristic curve analysis and estimation of the AUC. Since extubation failure is more common in extremely premature infants,^11,12^ further subgroup analysis was planned and performed in very prematurely born infants [22^+0^–28^+6^ completed weeks of gestation]. Statistical analysis was performed using the SPSS software version 26.0 (SPSS Inc., Chicago, IL).

## Results

**Fig. 1:**
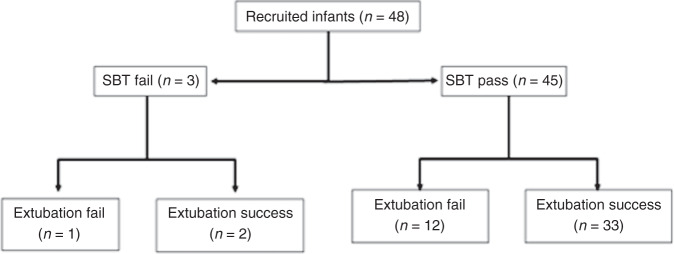
Flow diagram of the included infants, with those who failed the SBT on the left and those who passed the SBT on the right of the figure. Number of infants at each level shown in brackets.

Forty-eight infants (29 male) were recruited. The infants had a median (interquartile range) gestational age at birth of 27.2 (25.6–30.4) weeks and a weight of 940 (760–1115) g. The duration of mechanical ventilation was 4 (2–10) days. Infants were studied at a PMA of 30.2 (27.9–32.0) weeks and weight 1115 (853–1433) g [Table 1]. Ten infants were receiving 5 µg of intravenous morphine at the time of study of whom three failed extubation, but this was not associated with extubation failure (*p* = 0.982) [Table 1]. Three infants did not pass the SBT (6.3%) and 13 infants (27.1%) failed extubation. The result of the SBT was not associated with subsequent extubation success or failure (*p* = 0.801) [Fig. 1].

**Table 1:** Demographic and ventilatory parameters for all infants, showing differences in variables between those who were successfully extubated and those who failed.

	All infants (*n* = 48)	Extubation success (*n* = 35)	Extubation failure (*n* = 13)	*p* value
Gestational age (weeks)	27.2 (25.6 to 30.4)	27.7 (25.6 to 31.3)	27.0 (26.0 to 27.8)	0.417
Birth weight (g)	940 (760 to 1115)	935 (690 to 1185)	970 (765 to 1090)	0.808
Male gender	29 (60.4)	23 (65.7)	6 (46.2)	0.218
Postmenstrual age (weeks)	30.2 (27.9 to 32.0)	31.3 (29.0 to 33.7)	28.0 (26.9 to 28.6)	0.004
Duration of mechanical ventilation (days)	4 (2 to 10)	4 (2 to 9)	5 (2 to 10)	0.967
Fraction of inspired oxygen pre study	0.25 (0.21 to 0.30)	0.23 (0.21 to 0.30)	0.25 (0.23 to 0.29)	0.280
Haematocrit pre study (%)	41.1 (38.9 to 49.2)	42.8 (39.1 to 52.8)	40.8 (38.2 to 44.1)	0.164
Mean airway pressure pre study (cmH_2_0)	8.4 (7.0 to 10.3)	8.3 (6.9 to 9.9)	9.6 (7.8 to 10.9)	0.244
Partial pressure of carbon dioxide pre study (kPa)	5.6 (4.7 to 6.3)	5.3 (4.7 to 6.3)	5.7 (4.7 to 6.8)	0.560
Caffeine pre study (Y)	39 (88.6)	27 (87.1)	12 (92.3)	0.619
Sedation at time of study (Y)	10 (21.7)	7 (21.2)	3 (23.1)	0.982
Spontaneous breathing trial (SBT) pass	45 (94)	33 (94.3)	12 (92.3)	0.801
% change EMG amplitude (µV)	32.8 (10.3 to 81.6)	29.6 (8.4 to 85.2)	35.9 (14.2 to 52.2)	0.618
% change EMG_AUC_ (µV.S)	7.4 (−5.2 to 38.2)	3.4 (−10.0 to 24.6)	31.8 (6.4 to 55.6)	0.019

**Fig. 2:**
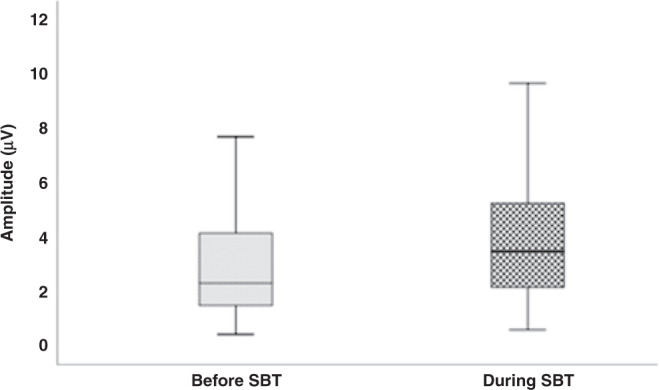
Amplitude before and during SBT. Amplitude in microvolts on the *y*-axis with the end of each box depicting the upper and lower quartiles, with the median marked by a horizontal line within the box. The whiskers mark the minimum and maximum values of amplitude.

The amplitude of the EMG increased during the SBT [2.3 (1.5–4.2) versus 3.5 (2.1–5.3) µV; *p* < 0.001] as did the EMG_AUC_ [3.9 (3.0–5.9) versus 4.8 (3.1–6.8) µV.S; *p* = 0.005] [Fig. 2]. The median increase of EMG_AUC_ during SBT was higher in those who failed extubation (31.7%) compared to those who succeeded extubation (3.4%; adjusted *p* = 0.038) and the median PMA was lower in those who failed extubation (28.0 weeks) compared to those who succeeded extubation (31.3 weeks; adjusted *p* = 0.012). Within the whole cohort of infants, the PMA was the strongest predictor for extubation failure (AUC 0.77), with a PMA of <29^+0^ weeks predicting extubation failure with 75% sensitivity and 85% specificity. In the whole cohort the percentage increase of EMG_AUC_ during SBT predicted extubation failure with an AUC of 0.61.

**Fig. 3:**
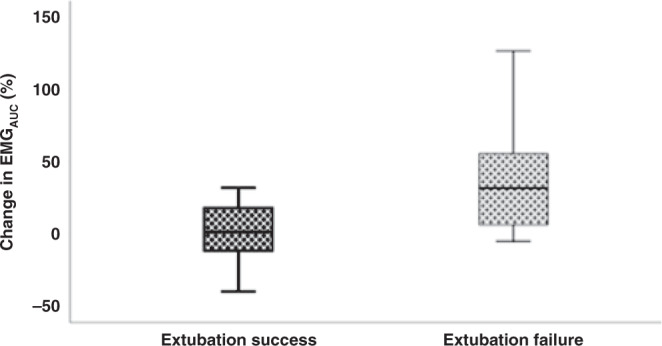
Change in EMG_AUC_ in less mature infants, showing the difference between those who fail extubation and those succeed. Change in % EMG_AUC_ on the *y*-axis with the end of each box depicting the upper and lower quartiles, with the median marked by a horizontal line within the box. The whiskers mark the minimum and maximum values of EMG_AUC_.

**Fig. 4:**
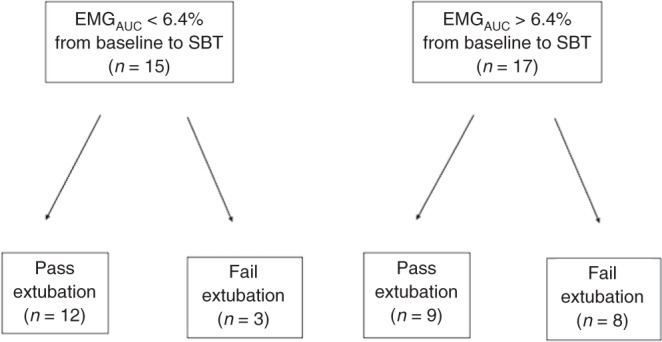
Flow diagram of infants <29 weeks who passed and failed extubation according to percentage change EMG_AUC_. Number of infants at each level shown in brackets.

In the subgroup of infants with a gestational age of <29 completed weeks, PMA (*p* = 0.038) and % change EMG_AUC_ (*p* = 0.027) were both associated with extubation failure [Fig. 3]. After correcting for variables that were different between extubation success and failure in the less mature cohort, the % change EMG_AUC_ (adjusted *p* = 0.035) remained the only factor independently associated with extubation failure (birth weight adjusted *p* = 0.080, PMA adjusted *p* = 0.058) [Table 2]. A % change EMG_AUC_ during the SBT of >6.4% predicted extubation failure with 73% sensitivity and 58% specificity (AUC 0.74) [Fig. 4].

**Table 2:** Differences in extubation failure of those less mature infants (22^+0^–28^+6^).

	Less mature infants who pass extubation (*n* = 21)	Less mature infants who fail extubation (*n* = 11)	*p* value
Gestational age (weeks)	25.9 (24.2 to 27.2)	26.9 (25.7 to 27.6)	0.411
Birth weight (g)	790 (655 to 973)	970 (760 to 1080)	0.051
Postmenstrual age (weeks)	30.3 (26.9 to 33.1)	27.9 (26.3 to 28.1)	0.038
Duration of mechanical ventilation (days)	11 (3 to 45)	7 (1 to 11)	0.203
SBT pass (Y)	19 (90.5)	10 (90.9)	0.968
% change EMG amplitude (µV)	26.9 (9.1–48.1)	35.9 (13.2 to 48.0)	0.815
% change EMG_AUC_ (µV.S)	1.54 (−12.0 to 21.4)	31.8 (5.5 to 55.8)	0.027

## Discussion

We have demonstrated that PMA was the best predictor of subsequent extubation failure in preterm infants but that in the subgroup of infants born <29 weeks of gestational age, the best predictor was derived from the diaphragmatic electromyogram. EMG has been successfully used to predict extubation in adults and children.^20,24,27^ In those populations, diaphragmatic impairment is associated with comorbidities, such as prolonged ventilation or coexisting infection.^37^ Preterm infants are affected by reduced diaphragmatic mass as they are in variable stages of diaphragmatic maturity,^38^ thus making our composite diaphragmatic assessment appropriate in determining the outcome of extubation in premature infants. The addition of assessment of the diaphragmatic EMG to the SBT could be used as an adjunct tool to aid assessment for readiness for extubation.

The increased amplitude of the diaphragmatic EMG during the SBT reflects the expected increase in respiratory effort that is needed to compensate for the reduction in the level of ventilatory support. An increase in Edi in response to a period of Et-CPAP has been reported in a cohort of 13 ventilated preterm infants with a lower median gestation than our cohort.^39^ Furthermore, the finding of increased diaphragmatic electrical activity in response to a lower level of ventilation was supported by a study that reported increased diaphragmatic electrical activity during weaning of preterm infants from high flow nasal cannula to no respiratory support.^40^

We should note that, although our study included preterm infants up to 37 weeks of gestation, extubation failure is more common in extremely preterm infants. Since this is the first study of predicting extubation in preterm infants with surface EMG during a SBT, we chose to study this phenomenon in the broader preterm population and include all preterm infants rather than only the extremely preterm ones. Our subgroup analysis points towards an increased utility of the test in the ones born extremely preterm and further studies could thus focus in this subgroup. We should also note that in our study we chose a 10-min duration for the SBT rather than a longer or a shorter duration. A longer duration might be associated with more extubation failure via inducing diaphragmatic fatigue from breathing against high resistance for a prolonged period^41^ while a very short duration might not had been sufficient to reveal sufficient changes in the EMG that would adequately predict the outcome of extubation.

The larger increase in EMG_AUC_ during the SBT in those of a lower gestational age (22^+0^–28^+6^) who went on to fail extubation is supported by results extrapolated from adult studies.^22,42,43^ One study, which assessed the relationship between neuro-ventilatory efficiency and extubation success, found ventilated adults who subsequently failed extubation had twice the increase in electrical activity of the diaphragm compared to those that who were successfully extubated.^22^ The EMG_AUC_ is an integration value describing the total amount of muscle activity during a given duration,^44^ and the increase may be reflective of increased tonic activity of the diaphragm suggesting loss of lung volume post extubation requiring more effort to recruit de-recruited areas of the lungs. Our results showed relatively poor performance of the SBT alone in predicting extubation failure, with more infants passing the SBT than failing extubation. The added value of the EMG is that it can be utilised to identify infants that pass the SBT but subsequently fail extubation. Following EMG analysis, attempts to extubate these infants would then be postponed. As our study, however, was not powered to detect these differences in the population of infants of <29 weeks of gestation, our results should be replicated in other centres in this specific population before direct clinical implementation.

Within the whole cohort, infants who were successfully extubated were of a higher PMA than those who failed. Some previous studies have reported a higher PMA to be predictive of extubation failure,^8^ with the authors suggesting that it is the prolonged period of ventilation prior to extubation that causes disuse atrophy of the diaphragm and thus subsequent extubation failure.^7,45^ Within our study, there was no difference, however, in the duration of invasive mechanical ventilation prior to extubation between those who were successfully extubated and those infants who were not. Indeed, our finding that a higher PMA is associated with successful extubation has been demonstrated in other previous studies and may also be linked to such infants having greater brain maturity, the development of which is crucial in controlling and regulating breathing.^4,6,26,46^

Other than changes in diaphragmatic activity during a SBT, changes in tidal volume and minute ventilation might also have held some prognostic value in predicting the outcome of extubation. A study of 41 preterm infants studied during a 2-h period of Et-CPAP before extubation described that the spontaneous expiratory minute ventilation was significantly lower in the infants who failed extubation compared to the ones who were successfully extubated.^47^ In our study, however, we did not incorporate measurements of tidal volume in order to avoid inserting an external flow sensor, which would be essential for accurate flow and volume measurements but would inadvertently increase the dead space and the resistance of the respiratory circuit and could thus potentially contribute to extubation failure. This study has strengths and some limitations. One of the strengths of this study is that the method of recording transcutaneous diaphragmatic EMG during an SBT is well tolerated by the preterm infants, with no associated adverse outcomes.^32,34,48^ Transcutaneous diaphragmatic measurement is simple to perform and can be carried out at the infant’s cotside with easy-to-use equipment, increasing the ability to predict clinical outcomes. Indeed, other techniques of diaphragmatic EMG measurement, such as by oesophageal EMG catheters [one single-use Edi catheter costs £145], are expensive in comparison to the system we utilised [EMG electrodes for ten infants cost <£10]. One limitation is that the transcutaneous diaphragmatic EMG recording can be influenced by infant movement, hence we only studied infants when no nursing cares were due. Furthermore, if spontaneous movements did occur, only stable sections of the EMG trace unaffected by large movement artefact were chosen for analysis. A second limitation is that our sample size was calculated from a study utilising oesophageal catheters to measure diaphragmatic activity, with results clearly being higher in voltage, and thus the potential influence of this different technique should be acknowledged. The increase in EMG during an SBT being a better predictor of extubation failure than gestational age in our cohort of infants born at <28 weeks gestation may reflect extreme prematurity within this subgroup. Use of a 48 h cut off for extubation failure in our study may miss the true number of extubation failures related to respiratory causes as has been suggested by previous authors.^11,13^ Furthermore, our specificity of the EMG results in predicting extubation failure was 58%, which means that a substantial number of infants will remain ventilated when they could have been otherwise successfully extubated. This low specificity, however, is a universal problem in the prediction of extubation outcome in premature infants as has been previously described.^14^ We should also acknowledge that our results are based on our unit local practice and might be influenced by our specific use of post extubation support and extubation criteria.

In conclusion, we have demonstrated that the electrical activity of the diaphragm in ventilated, preterm infants increases during a period of Et-CPAP. Furthermore, although in our cohort of premature infants the PMA was the best predictor of subsequent extubation failure, in those born at a gestational age lower than 29 weeks the increase in the diaphragmatic electrical activity was the best predictor of extubation failure with moderate sensitivity and specificity.

## Data Availability

The data sets generated during and/or analysed during the current study are available from the corresponding author on reasonable request.
